# Electrocortical Sources Related to Whole-Body Surface Translations during a Single- and Dual-Task Paradigm

**DOI:** 10.3389/fnhum.2016.00524

**Published:** 2016-10-18

**Authors:** Mark D. Bogost, Pablo I. Burgos, C. Elaine Little, Marjorie H. Woollacott, Brian H. Dalton

**Affiliations:** ^1^Department of Human Physiology, University of OregonEugene, OR, USA; ^2^Department of Kinesiology, Universidad de ChileSantiago, Chile; ^3^Faculty of Kinesiology, University of CalgaryCalgary, AB, Canada

**Keywords:** posture, standing balance, EEG, EEG/ERP, balance control, independent component analysis

## Abstract

Appropriate reactive motor responses are essential in maintaining upright balance. However, little is known regarding the potential location of cortical sources that are related to the onset of a perturbation during single- and dual-task paradigms. The purpose of this study was to estimate the location of cortical sources in response to a whole-body surface translation and whether diverted attention decreases the N1 event-related potential (ERP) amplitude related to a postural perturbation. This study utilized high-resolution electroencephalography in conjunction with measure projection analysis from ERPs time-locked to backwards surface translation onsets to determine which cortical sources were related to whole-body postural perturbations. Subjects (*n* = 15) either reacted to whole-body surface translations with (dual task) or without (single task) performing a visual working memory task. For the single task, four domains were identified that were mainly localized within the frontal and parietal lobes and included sources from the prefrontal, premotor, primary and supplementary motor, somatosensory and anterior cingulate cortex. Five domains were estimated for the dual task and also included sources within the frontal and parietal lobes, but the sources also shifted to other locations that included areas within the temporal and occipital lobes. Additionally, mean absolute N1 ERP amplitudes representing the activity from similar locations in both tasks were greater for the single than dual task. The present localization results highlight the importance of frontal, parietal and anterior cingulate cortical areas in reactive postural control and suggest a re-allocation or shift of cortical sources related to reactive balance control in the presence of a secondary task. Thus, this study provides novel insight into the underlying neurophysiology and contribution of cortical sources in relation to the neural control of reactive balance.

## Introduction

Balance and postural equilibrium are achieved through continuous integration and processing of sensory signals related to the orientation of our bodies and limbs in space (Massion, 1994, 1998). Indeed, cortical activity is likely important in maintaining upright postural equilibrium following whole-body perturbations (Dietz et al., 1985; Quant et al., 2004b; Little and Woollacott, 2015). However, little is known as to the precise cortical structures that are active during reactive balance control.

Appropriate reactive motor responses to translational movement are essential in maintaining upright balance. Short and longer latency muscle responses from the spinal cord and brain stem provide means of stabilizing the body immediately following a postural disturbance (Dietz et al., 1985; Ackermann et al., 1986; Macpherson and Inglis, 1993). Cortical potentials arising from a postural perturbation are thought to be involved in error detection for postural control; they would thus be used to update the central nervous system regarding the need to modify responses to upcoming perturbations, in order to impact subsequent control of upright equilibrium (Adkin et al., 2006). Conventional electroencephalography (EEG) allows for the characterization of event-related potentials (ERPs) that can be recorded during whole-body postural perturbations (Dietz et al., 1985; Ackermann et al., 1986; Staines et al., 2002; Quant et al., 2004b; Maki and McIlroy, 2007). Typical ERP components (i.e., P1, N1 and P2) that appear following a rapid surface translation task are thought to represent kinesthetic feedback from the peripheral limbs (Dietz et al., 1985; Ackermann et al., 1986; Staines et al., 2002; Quant et al., 2004b; Maki and McIlroy, 2007). Moreover, the N1 latency in particular is useful in characterizing cortical processing of sensory dynamics due to its consistent appearance compared with the other components (Quant et al., 2004b; Adkin et al., 2006; Little and Woollacott, 2015). Most previous studies investigating ERPs in response to whole-body postural perturbations selected specific electrode locations at the scalp and did not attempt to locate the cortical sources related to maintaining reactive postural control.

Furthermore, dual-task paradigms require simultaneous engagement with a cognitive and motor task (Lajoie et al., 1993; Vander Velde and Woollacott, 2008). Previous studies have demonstrated how various cortical areas likely assist with postural equilibrium due to decrements in performance in either cognitive or postural tasks and an attenuated N1 ERP amplitude (Quant et al., 2004a; Adkin et al., 2006; Little and Woollacott, 2015), ostensibly owing to a finite capacity of the cortex to perform parallel processing (Wickens, 1983). A limited number of investigations have examined cortical activity time-locked to whole-body surface translations when participants performed a cognitively demanding task (Quant et al., 2004a; Adkin et al., 2006; Little and Woollacott, 2015). For example, Adkin et al. (2006), utilized a visual tracking task during whole-body surface translations and found decrements in both tracking performance and N1 amplitude beneath the frontocentral electrode (i.e., Fz and Cz) locations compared with a reactive standing task alone. Further, Little and Woollacott (2015) corroborated these findings by using a visual working memory (VWM) task during whole-body surface translations and reported a decrease in the N1 amplitude beneath electrodes positioned over the motor and somatosensory cortical areas during a dual-task paradigm compared with a single task. These aforementioned reports provide evidence that attentional and postural systems are in competition within the cortex to maintain upright balance, but do not provide insight regarding the cortical sources contributing to the N1 ERP nor the re-allocation of resources responsible for its attenuation during a dual task.

Methodological improvements allow investigation into the electrocortical dynamics during whole-body motion that likely includes head movements, such as perturbations, walking and even running (Makeig et al., 2009; Slobounov et al., 2009; Gramann et al., 2010; Gwin et al., 2010; Sipp et al., 2013; De Sanctis et al., 2014; Seeber et al., 2015). High-density EEG in conjunction with independent component analysis (ICA) and source localization methodology can be used to estimate electrocortical areas that are synchronized to related events (Gwin et al., 2010; Wagner et al., 2012, 2014) and to discriminate these from non-cortical artifacts such as eye and muscle activity (Gramann et al., 2010; Gwin et al., 2010). Recently, investigators have attempted to determine the location of cortical activity related to upright postural control (Slobounov et al., 2009; Marlin et al., 2014). For example, one study (Slobounov et al., 2009) illustrated cortical activity within the anterior cingulate cortex (ACC), the limbic area, and at the junction of the precuneus and occipital lobe when human subjects stood on one leg with their eyes closed. The authors suggested these cortical structures may assist with predicting future postural instability and were activated due to the unstable nature of the task. Because these results are inconsistent with previous findings related to upright bipedal posture (Marlin et al., 2014), additional studies are required to locate the cortical sources related to reactive balance control.

The purpose of this study was to estimate the location of the cortical sources related to a whole-body surface translation during a single- and dual-task paradigm. A second aim was to determine whether a secondary cognitive task (i.e., diverted attention) attenuated the N1 ERP amplitude corresponding to the postural perturbation. We hypothesized that electrocortical activity related to the whole-body surface translations would be located within the sensory and (pre)motor cortices and the dual task would elicit additional electrocortical sources within the frontoparietal attentional networks, similar to previous work using functional magnetic resonance imaging (fMRI; Deprez et al., 2013; Johannsen et al., 2013). We also expected that during a dual-task compared with a single-task paradigm, the N1 ERP amplitude would decrease within the cortical areas related to the reactive postural task.

## Materials and Methods

### Subjects

Fifteen young adults (13 females and 2 males) with no history of concussion, color-blindness or psychiatric conditions were recruited for this study. The participants in this study were taken from a subset of a larger study (Little and Woollacott, 2014, 2015) and ranged in age from 19 to 24 years (age: 20.3 ± 1.5 years; height: 166.8 ± 7.7 cm; mass: 69.6 ± 8.3 kg). This study was carried out in accordance with the recommendations of the University of Oregon’s Institutional Review Board, the Committee for the Protection of Human Subjects with written informed consent from all subjects. All subjects gave written informed consent in accordance with the Declaration of Helsinki. The protocol was approved by the University of Oregon’s Committee for the Protection of Human Subjects (protocol #:06222011.085).

### Visual Working Memory Task

**Figure 1:**
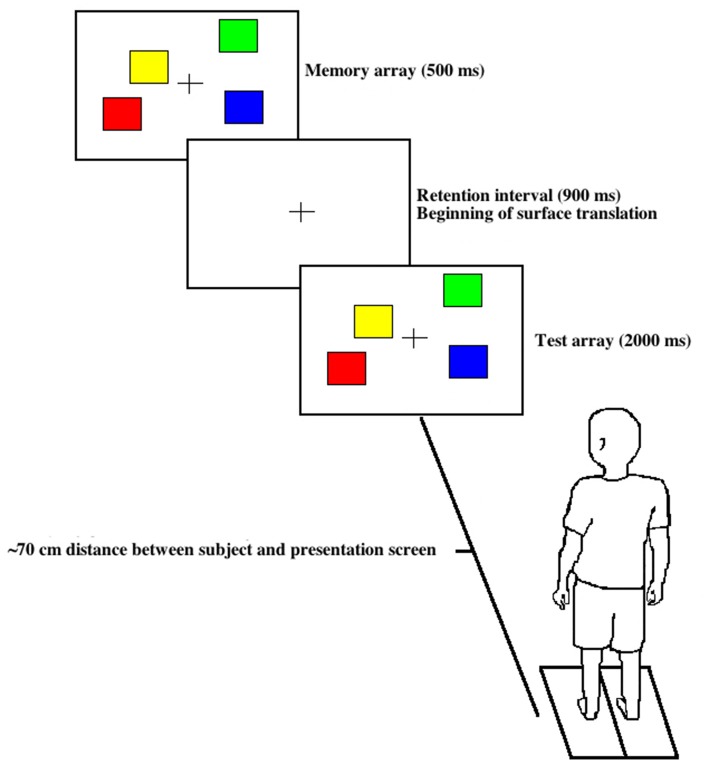
**Visual working memory (VWM) task.** A memory array was presented first for 500 ms with a distinct color and spatial configuration followed by a 900 ms consolidation phase with a concurrent surface translation followed by a 2000 ms test array presentation, either congruous or incongruous with the original memory array.

The change detection task to assess VWM was implemented for this study (Pashler, 1988; Vogel and Machizawa, 2004; Little and Woollacott, 2015). All subjects were familiarized with the protocol to remove the possibility of any learning effects. The VWM change task involved subjects viewing a memory array of 2, 4 or 6 squares for 500 ms that were presented on a screen ~70 cm away. Immediately following this, a blank screen with a central cross was shown for 900 ms (retention interval), during which time subjects stored the number, position and color of arrays (Figure 1). Immediately following the retention interval, a test array was presented for 2000 ms where subjects were instructed to determine if the memory array presented was congruous or incongruous with the test array, using a bilateral button press (left press = similar, right press = different). For visual representation please see Figure 1A in Little and Woollacott (2015). The dual-task condition consisted of the presentation of three sets of squares (30 trials for each) for a total of 90 trials, which included 2, 4 and 6 square arrays. However, only the 4 square arrays (moderate level of difficulty) were used for analysis to match the number of trials collected for the single task. Square colors (red, blue, purple, green, yellow, black and white) were presented randomly throughout all trials. No more than two identical colors were presented within each memory and test array. Subjects were instructed to pay attention to a cross placed in the center of the screen (Figure 1) for all trials to ensure that visual attention was consistent and gaze was controlled for the single and dual tasks.

### Surface Translations

Subjects stood quietly on a surface translation platform with a comfortable stance. The position of their feet was traced with masking tape to serve as a reference, if any stepping occurred. If a subject stepped, that trial was discarded from the analysis and they were instructed to move back to the previously traced position. A hydraulically driven platform with a displacement amplitude of 10 cm, peak velocity of 30 cm/s, 500 ms duration and acceleration of 0.34 m/s^2^ was used for this protocol.

Subjects were instructed to pay equal attention to the VWM task and maintain their neutral stance position. Backward surface translations with the same amplitude and velocity were delivered with and without the VWM task (dual and single-task conditions, respectively). To minimize temporal anticipation of the surface translations, inter-trial intervals were varied between 8–15 s. Randomized forward surface translations were also delivered intermittently to promote upright posture and prevent anticipation of the translational perturbation characteristics. During the dual-task trials, backward surface translations were time-locked to occur immediately following the memory array (Figure 1). To counter-balance the experimental paradigm, 14 and 11 single-task trials were inserted before and after all dual-task trials, respectively. Even though 14 single-task trials always preceded the dual-task condition, adaptation is likely not a factor here, as participants exhibited greater area under the center of pressure trajectory, AP force and peak center of pressure trajectory for the dual task than single task (Little and Woollacott, 2014, 2015). Subjects were instructed to randomly trigger the button press during the single-task trials to ensure motor requirements were equivalent for both tasks. The subjects were fitted with a harness attached to an overhead trolley to provide safety throughout the protocol. Because there was a significant reduction in VWM capacity and reactive postural control (increase in peak center of pressure trajectory) for the dual task compared to control (sitting and the single task, respectively; Little and Woollacott, 2015), the dual-task paradigm incorporated here was sufficient to induce cognitive interference between the VWM task and reactive postural control.

### Electroencephalography Data Collection

Continuous EEG was recorded using a Hydrocel Geodesic Sensor net with 256 electrodes (Electrical Geodesics Inc., Eugene, OR, USA). A central electrode (Cz), located midway between the nasion and inion and positioned midway between the preauricular points, was used to reference 255 channels. Hydrocel caps were soaked in an electrolyte solution to ensure proper conduction at the scalp. As recommended by the manufacturer (Electrical Geodesics Inc., Eugene, OR, USA) and used previously (Goodin et al., 2012; Kashihara, 2014; Little and Woollacott, 2015), the electrode impedance was kept below 50 kΩ throughout testing. The amplifier used here is designed for higher electrode impedances and the maintenance of recording accuracy. All data were sampled at 1 kHz and amplified ×2000 (Net Amps 300 amplifier, Electrical Geodesics, Eugene, OR, USA). The data were collected for a previous study (Little and Woollacott, 2015), but analyzed here using different techniques and hypotheses to generate novel results.

### Electroencephalography Data Analysis

**Figure 2:**
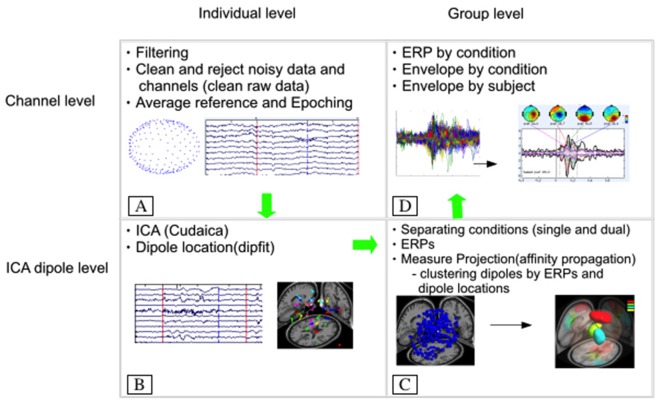
**Electroencephalography (EEG) data processing flow chart. (A)** Represents the initial filtering, cleaning and referencing of the data, which is followed by decomposition of the data using independent component analysis (CUDAICA) and localization of each dipole for each subject **(B)**. Next, each condition (dual and single task) was separated and measure projection analysis was applied to cluster similar dipoles by their respective event-related potentials (ERPs) and location within the cortex **(C)**. Last, **(D)** represents the back projection of data from the dipole to the electrode creating grand average N1 ERPs and envelopes by condition for all subjects.

All EEG data were analyzed using customized MATLAB (The Math Works Inc. Natick, MA, USA) scripts generated from the open source EEGLAB^1^ (Schwartz Center for Computational Neuroscience, La Jolla, CA, USA). For a visualization of the data analysis process see Figure 2. Data were high- and low-pass filtered digitally at 1 and 58 Hz, respectively.

All artifacts were removed from the channels’ data utilizing the artifact subspace reconstruction model from EEGLAB. Artifact subspace reconstruction rebuilt data using a spatial mixing matrix, removing signals with high variability, assuming volume conduction. An average reference was applied to the data. ICA was performed on 255 original channels to separate EEG signals into static, temporally independent components (Bell and Sejnowski, 1995; Liao et al., 2005). CUDAICA (optimized Infomax ICA algorithm) was used on continuous data decomposing the information into independent components. Afterward, epochs for the ERPs were generated with an 1100-ms window time-locked to the onset of the surface translation (range: −400 to 700 ms). The ERP data were filtered at 25 Hz, corrected with a baseline from −400 to 0 ms and averaged across for the single and dual task. Dipoles were then estimated utilizing DIPFIT from the open source code available. We applied a rejection threshold such that dipoles exhibiting less than 15% residual variance between the actual independent component scalp map and the model projection of the equivalent dipole to the same electrode montage were kept for further analysis (Onton et al., 2006).

The statistically based clustering method implemented for this study, using the decomposed EEG data (i.e., ERPs) following ICA and source estimations, determined the similarities between subjects for both single- and dual-task conditions. Specific trials used for processing included those with both surface translations and VWM task (dual task) and those with surface translations only (single task). Brain dipole clustering was achieved using measure projection analysis through use of the measure projection toolbox (MPT^2^: Schwartz Center for Computational Neuroscience) outlined in detail by Bigdely-Shamlo et al. (2013). Briefly, measure projection analysis cluster components based on the ERP and dipole locations of data exhibited statistically significant consistency (e.g., dipolarity, amplitude, latency) for all subjects for each condition. Mean EEG values were then assigned to all cortical locations based on a Gaussian density. A three-dimensional Gaussian location error equal to 12 mm with three standard deviations (36 mm) was applied to data for location estimations. Measure projection calculates the expected value *E{M(y)}* of the measure, *M* at brain locations spanning a grid of ~8 mm spacing:

(1) $$E\left\{M\left(y\right)\right\}\;=\;\left\{M\left(y\right)\right\}\;=\;\frac{\sum_{i\;=\;1}^{n}P_{i}\left(y\right)M_{i}}{\sum_{i\;=\;1}^{n}P_{i}\left(y\right)}$$

Afterward, measure projection obtains significance values for the cortical locations to determine which areas have similarities between dipoles. This is accomplished via calculating convergence *C*(*y*) at each location:

(2) $$C\left(y\right)\;=\;E\left\{S\left(y\right)\right\}\;=\;\frac{\sum_{i\;=\;1}^{n}\sum_{j\;=\;1,j\;\neq \;i}^{n}P_{i}\left(y\right)P_{j}S_{i,j}}{\sum_{i\;=\;1}^{n}\sum_{j\;=\;1,j\;\neq \;i}^{n}P_{i}\left(y\right)P_{j}\left(y\right)}$$

In the convergence equation above, *P*_i_(*y*) is the probability density of dipole *i* at cortical location *y* and *S*_i,j_ is the correlation between vectors associated with dipoles *i* and *j* (Bigdely-Shamlo et al., 2013). Voxels outside of the Montreal Neurological Institute (MNI) template were excluded from analysis. Lastly, domains reflecting probable source resolved activity were constructed based on the correlation between ERPs within adjacent areas. Parameters included a correlation threshold of 0.7 with a *p* < 0.05 and measure projection analysis was applied to surrogate data with a false discovery rate correction with *p* ≤ 0.012. A time-wise threshold detecting significant points was created utilizing 2000 surrogate convergence values at each voxel related to the null hypothesis that no association exists between brain locations and ERPs. Conditions analyzed included single and dual tasks separately and both single and dual tasks combined.

After dipoles and domains were computed, we returned to the channel level (single- and dual-task conditions combined) by performing a back projection analysis from individual ICA sources (clustered in domains) to activity at the electrode. Analysis of both conditions combined was performed to compare the differences between conditions because separate domain locations were observed for the single and dual tasks. All ERP statistical processing was performed on EEGLAB 13.2 software. ERP envelopes were used to examine the most prominent positive and negative channel values at each time point and to find cortical domains with the highest variability in the envelope’s epoch (−400 to 700 ms). Additionally, a smaller time window between 50–190 ms was applied to all epochs focusing on the N1 ERP for dual and single-task conditions.

Percent variance accounted for (pvaf) was also calculated using:

(3) $$PVAF\;=\;100\times \left(1-\frac{Var\left(D-B\right)}{VarD}\right)$$

with *D* and *B* representing channel data and back projection of each brain domain onto scalp channels respectively. The pvaf values illustrate how much variability of the grand average ERP is explained based on source activities (domain dipoles) within the brain, thus providing a better representation of the cortical areas contributing to the grand average compared with measured ERP amplitudes at electrode sites on the scalp. The calculated pvaf values may exceed 100% as calculated scalp projections are spatially correlated, not orthogonal. As such, projections may cancel one another out. ERP envelopes were constructed using the EEGLAB plugin std_envtopo, utilizing cluster contributions plotting envelopes for every condition. The statistical analysis applied to this study was used to understand the differences between single and dual-task conditions for each comparable brain domain area.

In order to compare the absolute ERP amplitude for single and dual-task conditions an automatic algorithm was used to estimate the absolute peaks for the envelopes in both conditions (from 90 to 170 ms). The envelope activity was obtained after a back projection only from domains 1 to 4 in the combined analysis, excluding other dipole activities that did not belong to these domains. The absolute values were used because we did not assume that the N1 was always a negative activity in all the channels. This is based on the assumptions that: (1) dipole polarities can be different among subjects; and (2) the average reference can change the polarity of the channel’s ERP. It is important to consider subject variability for the N1 peak latencies, which can create differences between the individual values and averaged values for peaks. Differences were assessed using a paired *t*-test and data are reported as means ± 95% confidence intervals.

## Results

**Figure 3:**
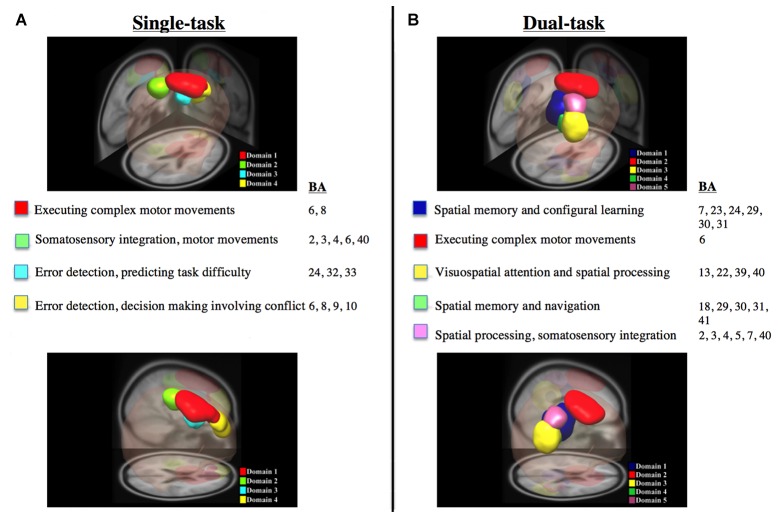
**Dipole data derived from measure projection analysis of all subjects for the single and dual-task datasets.** **(A,B)** Illustrate posterior and lateral viewpoints of significant domains along with their respective Brodmann areas (BAs) and functions. All data was derived from dipole data using measure projection analysis for all subjects for single and dual-task datasets, respectively.

The measure projection analysis clustering algorithm was used to provide estimates of distinct cortical sources related to the single and dual-task conditions for all subjects (Figure 3). Anatomical and Brodmann area (BA) probability values related to each domain are listed within Table 1 and calculated using the Laboratory of Neuroimaging project probabilistic (Department of Neurology, University of California, Los Angeles, CA, USA) and BA (Lancaster et al., 1997; Shattuck et al., 2008) atlases. Listed functional associations with each BA are based on Brodmann’s interactive atlas (Bigdely-Shamlo et al., 2013). The visual representations of cortical domains with larger volumes demonstrate increased physical distances for all dipoles and the opposite for those with more condensed volumes. For descriptions of BAs and anatomical locations distinct domains see Table 1. As a note, the methodologies used here estimated source localization within subcortical areas (e.g., caudate) related to the postural perturbation, but owing to technological concerns and limitations of EEG (Olbrich, 2015), we refrain from reporting and discussing these results within this manuscript.

**Table 1:** **Anatomical locations representing cortical domains generated with the respective Brodmann Areas (BAs), related probability of activity within each BA, and specific anatomical locations for the single and dual tasks for all subjects**.

	*Brodmann area*			*Anatomical area*	
Domain	Area	Prob.	Description	Area	Prob.

**Single task**					

1	BA 6	0.82	Premotor and supplementary motor	L Superior frontal gyrus	0.61
	BA 8	0.07	Lateral and medial supplementary motor areas	R Superior frontal gyrus	0.34
2	BA 3	0.30	Primary somatosensory	L Postcentral gyrus	0.49
	BA 4	0.23	Primary motor	L Precentral gyrus	0.49
	BA 2	0.20	Primary somatosensory
	BA 40	0.17	Spatial processing
	BA 6	0.05	Premotor and supplementary motor
3	BA 24	0.64	Error detection	L Cingulate gyrus	0.60
	BA 32	0.24	Prediction of task difficulty	L Superior frontal gyrus	0.10
	BA 33	0.11	Error detection	R Cingulate gyrus	0.07
4	BA 9	0.47	Error processing and detection	L Middle frontal gyrus	0.71
	BA 10	0.35	Decision making involving conflict	L Superior frontal gyrus	0.25
	BA 8	0.10	Lateral and medial supplementary motor areas
	BA 6	0.06	Premotor and supplementary motor

	**Dual task**					

1	BA 31	0.44	Spatial memory and configural learning	L Cingulate gyrus	0.38
	BA 23	0.26	Spatial memory and configural learning	R Cingulate gyrus	0.33
	BA 7	0.08	Somatosensory association	R Precuneus	0.08
	BA 30	0.08	Spatial processing	L Precuneus	0.06
	BA 24	0.07	Error detection
	BA 29	0.06	Navigation, processing scenes
2	BA 6	0.87	Premotor and supplementary motor	L Superior frontal gyrus	0.56
				R Superior frontal gyrus	0.40
3	BA 39	0.44	Visuospatial attention	R Angular gyrus	0.65
	BA 40	0.20	Spatial processing	R Superior parietal gyrus	0.12
	BA 22	0.16	Auditory processing	R Supramarginal gyrus	0.09
	BA 13	0.08	Inferior insula	R Middle occipital gyrus	0.06
				R Middle Temporal gyrus	0.05
4	BA 31	0.51	Spatial memory and semantic processing	R Superior parietal gyrus	0.84
	BA 29	0.19	Navigation, processing scenes	R Angular gyrus	0.07
	BA 30	0.17	Spatial processing	R Parahippocampal gyrus	0.05
	BA 18	0.06	Secondary visual
	BA 41	0.05	Primary and auditory association
5	BA 40	0.30	Spatial processing	R Postcentral gyrus	0.49
	BA 3	0.17	Primary somatosensory	R Superior parietal gyrus	0.33
	BA 7	0.16	Somatosensory association	R Angular gyrus	0.07
	BA 5	0.11	Somatosensory association	R Precentral gyrus	0.06
	BA 4	0.11	Primary motor	R Supramarginal gyrus	0.05
	BA 2	0.08	Primary somatosensory

### Domain Results

**Figure 4:**
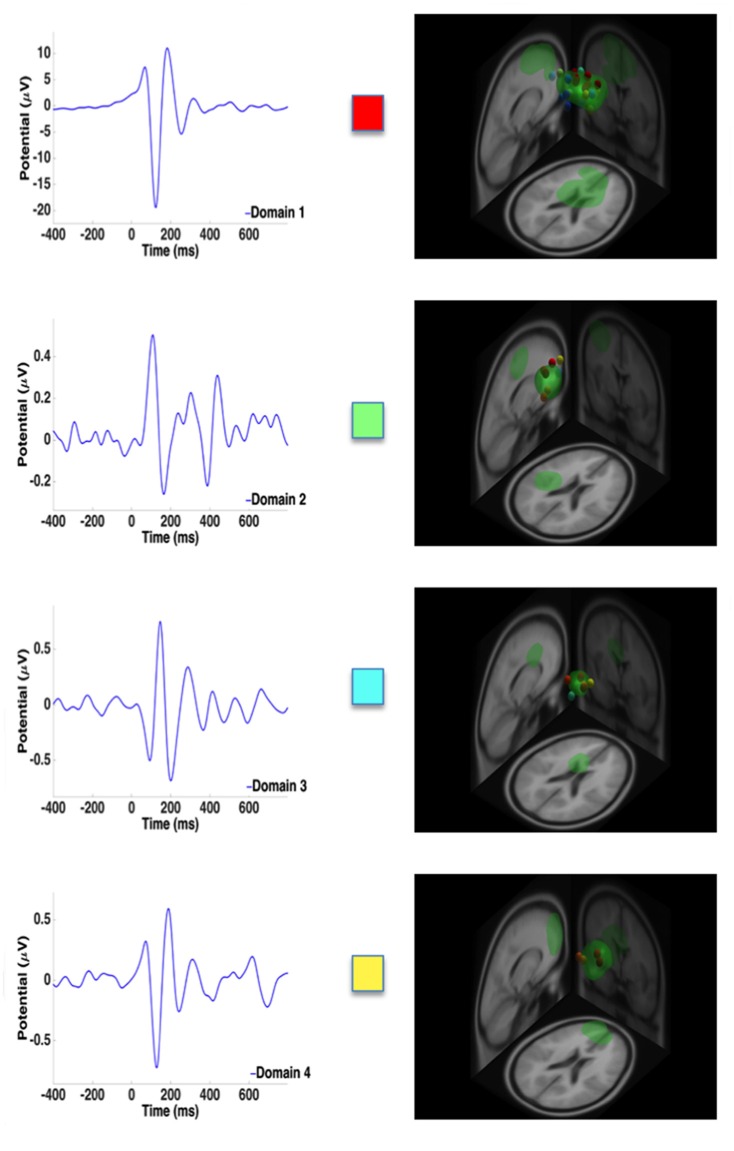
**Representative ERPs from each domain for the single task.** High contributing dipoles calculated from the measure projection analysis are also included. Domains are color coded to match domain areas represented in Figure 3A.

**Figure 5:**
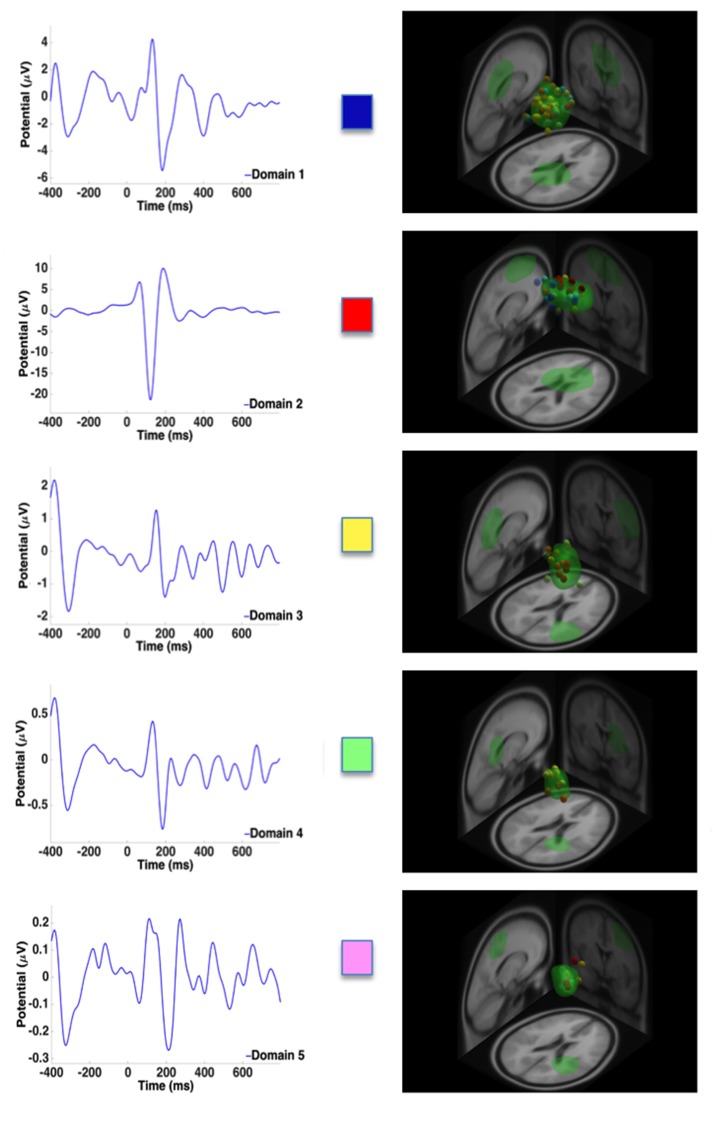
**Representative ERPs from each domain for the dual task.** High contributing dipoles calculated from the measure projection analysis are also included. Domains are color coded to match domain areas represented in Figure 3B.

ERPs along with the high contributing dipoles from measure projection analysis are provided in Figures 4 and 5 to illustrate the ERP shape and latency associated with distinct domains for the single and dual tasks, respectively. Overall, domain 1 and 2 ERP waveforms for the single and dual-task conditions, respectively, provided a much better signal to noise ratio than the other domains. These aforementioned domains, representing the premotor and supplementary motor areas, exhibited the largest N1 ERP amplitudes in comparison with other domains. As represented by domain 1, the greatest number of dipoles were observed within the spatial memory and learning areas (cingulate cortex) for the dual task and premotor and supplementary motor areas for the single task compared with other domains.

For the single task, the measure projection analyses constructed four domains in relation to the onset of the surface translation (Figure 3A; Table 1). Domain 1 and 4 were localized within the frontal lobe and consisted of premotor and supplementary motor areas and areas within the prefrontal cortex involving error processing and detection. Domain 2 was located within the frontal and parietal lobes and consisted of dipoles representative of the primary motor and somatosensory cortex; whereas domain 3 was localized to the ACC and frontal cortex. For the dual task, five domains were estimated in relation to the onset of the surface translations (Figure 3B, Table 1). Domain 1 represented cortical sources within the parietal and cingulate cortical areas and domain 2 was localized to the frontal cortex, mainly within the premotor and supplementary motor areas. Domain 3 consisted of contributing dipoles within the parietal, temporal and insular cortical areas. Domain 4 consisted of source localization within the occipital, temporal, and cingulate cortical areas; whereas domain 5 was located within the frontal (primary motor) and parietal (primary somatosensory and somatosensory association) lobes.

Because estimated domains were different between single and dual tasks, we combined all trials to compare attenuation of the N1 ERP amplitude in the areas that overlapped, which resulted in the generation of four distinct domains. Further description for each domain can be found in Table 2.

**Table 2:** **Anatomical locations representing cortical domains generated with the respective Brodmann areas (BAs), related probability of activity within each BAs, and specific anatomical locations for both conditions combined for all subjects**.

	*Brodmann area*			*Anatomical area*	
Domain	Area	Prob.	Description	Area	Prob.

**Combined**					

1	BA 6	0.85	Premotor and supplementary motor	L Superior frontal gyrus	0.58
	BA 8	0.05	Lateral and medial supplementary motor areas	R Superior frontal gyrus	0.38
2	BA 31	0.39	Spatial memory and configural learning	R Cingulate gyrus	0.55
	BA 23	0.35	Spatial memory and configural learning	L Cingulate gyrus	0.27
	BA 30	0.10	Spatial processing	R Precuneus	0.08
	BA 29	0.08	Navigation, processing scenes
	BA 24	0.06	Error detection
3	BA 39	0.49	Visuospatial attention	R Angular gyrus	0.73
	BA 22	0.19	Auditory processing	R Superior parietal gyrus	0.13
	BA 40	0.16	Spatial processing	R Supramarginal gyrus	0.06
	BA 13	0.07	Inferior insula
	BA 19	0.05	Associative visual (V3)
4	BA 40	0.39	Spatial processing	R Postcentral gyrus	0.66
	BA 5	0.27	Somatosensory association	R Superior gyrus	0.34
	BA 1	0.14	Primary somatosensory
	BA 2	0.14	Primary somatosensory
	BA 7	0.05	Somatosensory association

### ERP Envelopes

**Figure 6:**
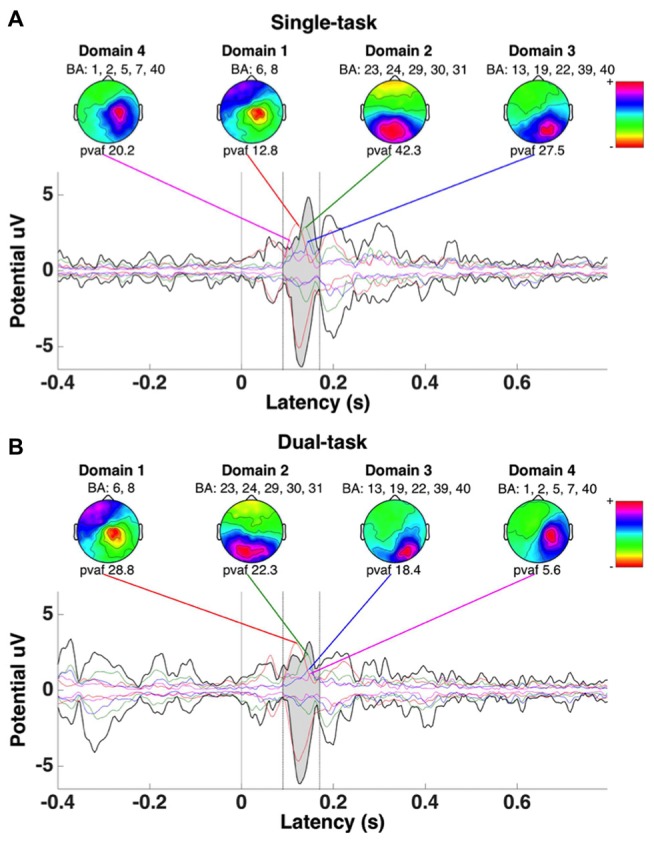
**ERP envelopes of a multichannel epoch developed using measure projection analysis for all subjects for the single (A) and dual tasks (B) taken from the combined data.** Thick dark lines represent minimum and maximum potential values for all channels. Mean percent variance accounted for (pvaf) measured between 90 and 170 ms, representing the N100 waveform from each respective domain area, is provided. Domains 1–4 correspond with those provided in Table 2.

**Figure 7:**
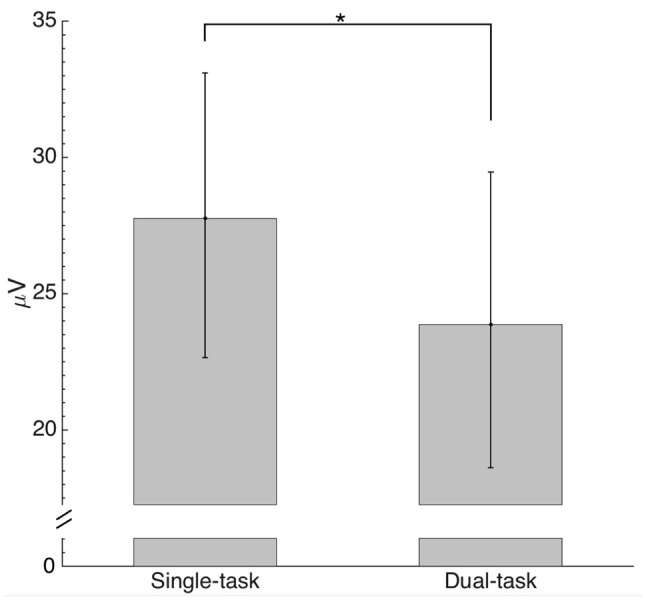
**Comparison of absolute maximum envelope values for the N1 back projected from estimated domains (individual peak values between 90 and 170 ms) by condition.** The asterisk indicates a significant difference (*p* < 0.05).

Activity under all electrode sites was analyzed utilizing ERP envelopes (Figure 6) differentiating between global and localized cortical regions by grand average ERP envelopes of all electrodes and calculated pvaf values from each domain respectively. These domains and pvaf values were derived from the combined analysis for both conditions. Only four domain regions accounting for the largest amount of variability in the data (pvaf values) were analyzed in this study, corresponding to Table 2. The pvaf values for the single task were substantially greater than the dual task for domains 2 (42.3 vs. 22.3%, respectively) with sources located within the anterior and posterior cingulate cortex, domain 3 (insular cortex and occipital, temporal and parietal lobe; 27.5 vs. 18.4%, respectively) and domain 4 (20.2 vs. 5.6%, respectively) with representative dipoles located within the parietal cortex (somatosensory areas); whereas domain 1 (premotor and supplementary motor cortex) depicted lesser pvaf values for the single than dual task (12.8 vs. 28.8%, respectively). The summation of pvaf values exceed 100% as scalp projections are spatially correlated, not orthogonal, leading to a cancellation of projections. As shown in Figure 6, analysis of electrode data, back projected from the four detected domains for the combined conditions revealed significant maximum N1 ERP amplitude attenuation for the dual compared with single task (*p* = 0.01; Figure 7).

## Discussion

The primary aim of the current study was to estimate the location of cortical areas related to the onset of whole-body surface translation with or without performing an executive function task (i.e., VWM task). Second, we aimed to address whether the N1 ERP amplitude was attenuated for the dual compared with single task within the same cortical regions. For the reactive balance task alone (single task), the domains identified related to the whole-body perturbation included cortical representation from primary, pre-, and supplementary motor, primary somatosensory and spatial processing and error detecting areas (Table 1). Moreover, for the dual task, cortical sources shifted to include visuospatial attention, but less error detection areas (Table 1). In agreement with our hypothesis, when N1 ERP amplitude from the four most representative domains was compared, there was a significant depreciation for the dual than single task (Figure 7). Our novel results provide convincing evidence regarding the cortical sources and attenuation of the N1 ERP in relation to reactive postural control and extend previous findings on upright posture (Dietz et al., 1985; Staines et al., 2002; Quant et al., 2004a; Adkin et al., 2006; Little and Woollacott, 2015; Mierau et al., 2015) and dual-task processing using fMRI (Deprez et al., 2013; Johannsen et al., 2013).

For the single-task condition, domains 1, 2 and 4 were estimated to include the somatosensory and pre-, primary and supplementary motor cortices (Table 1), which corroborate previous findings (Dietz et al., 1985; Quant et al., 2004b; Adkin et al., 2006; Marlin et al., 2014; Little and Woollacott, 2015; Mierau et al., 2015). Furthermore, domains 3 and 4 of the single task included cortical sources representing error detection and processing areas localized to the ACC and frontal cortex (Table 1). Previously, some studies (Slobounov et al., 2009; Mierau et al., 2015) reported the involvement of the ACC during a single-legged balance task, but another study (Marlin et al., 2014), implementing a lean and release reactive balance paradigm, did not. The disparate findings may be related to differences in experimental paradigms or difficulty of the respective reactive postural control tasks. Adkin et al. (2006) speculated that following unexpected perturbations, error-detecting centers are activated to assess overall postural state before and after translational movement to ensure that the appropriate motor responses are selected to achieve balance equilibrium. Thus, based on our results and that of others (Dietz et al., 1985; Quant et al., 2004b; Adkin et al., 2006; Slobounov et al., 2009; Marlin et al., 2014; Little and Woollacott, 2015; Mierau et al., 2015), the motor and somatosensory areas and ACC are likely important cortical sources related to reactive balance control.

Even though, many of the estimated cortical sources were similar for the single and dual tasks, notable differences between conditions were observed, particularly within the motor and error detection regions. For example, three cortical domains (1, 2 and 4) were identified for the single task in which motor areas were most likely associated with the corresponding domain (probability = 0.89, 0.28 and 0.16, respectively); whereas only two domains (2 and 5) were estimated for the dual task (probability values of 0.87 and 0.11, respectively; Table 1). Error detection and processing areas were represented by two domains (3 and 4) for the single task (Table 1); however, the dual task only had one source (domain 1), which exhibited a low probability that an error detection area (ACC) was most likely associated with the respective domain (Table 1). These results provide direct evidence in support of the idea of a limitation in the availability and re-allocation of cognitive resources while processing tasks simultaneously (Wickens, 1983; Little and Woollacott, 2015). During the present protocol, participants were fixated on a VWM task for the dual-task protocol and prior to the perturbation, their cognitive resources were likely distributed to posterior parietal and occipital cortex areas (Vogel and Machizawa, 2004; Xu and Chun, 2006; McCollough et al., 2007). Activating these particular areas prior to the perturbation onset would potentially withdraw cortical resources from the motor, somatosensory and error detection areas, limiting overall availability to efficiently process activity related to the postural perturbation. Our localization results in combination with significant decrements in the behavioral outcome measures (i.e., VWM capacity and center of pressure peak trajectory) for the dual compared with single task (Little and Woollacott, 2015) indicate a re-distribution and sharing of attentional resources.

In support of limited cognitive resources, analysis of activity over all electrode sites, as represented by the ERP envelopes, revealed attenuation of the N1 ERP amplitude for the dual compared with single task (Figure 7). This study supports and expands upon previous reports only analyzing N1 ERP amplitudes from pre-selected electrodes (Quant et al., 2004b; Adkin et al., 2006; Little and Woollacott, 2015) by providing calculated pvaf values for each domain. The pvaf values illustrate how much variability of the grand average ERP is explained based on source dynamics (domain dipoles) within the brain. Thus, this analysis may provide a better representation of the cortical areas contributing to the grand average compared with measured ERP amplitudes at the electrode sites; a technique traditionally performed in EEG analysis (Quant et al., 2004a; Adkin et al., 2006; Little and Woollacott, 2015).

In contrast to the reduced cortical sources associated with motor and error processing, there was an increase in the localization of areas involved with spatial processing and attention for the dual compared with single task. For the single task, only domain 2 included the probability that a spatial processing area was most likely associated with that source and was localized to the parietal cortex (Table 1); however, for the dual task, four sources (domains 1, 3, 4 and 5) consisting of spatial processing and attention areas (Table 1) were localized within the parietal and posterior cingulate cortices. It is interesting that spatial processing and attention are related to the onset of the perturbation and this may indicate that these areas are related to the simultaneous organization of visual-spatial change detection and the spatial coordination of reactive postural control during a dual-task paradigm. Previously, an fMRI study, aimed at elucidating the neural correlates mediating parallel processing of voluntary ankle motion and simultaneous executive function (Johannsen et al., 2013) reported that the inferior frontal gyrus likely facilitates dual-task processing; whereas another fMRI report found a large distribution of frontoparietal activity during concurrent auditory and visual cognitive tasks (Deprez et al., 2013). Similarly, for the dual-task condition in our study, event-related cortical sources were estimated within the frontal, parietal, cingulate, occipital and temporal cortices, indicating several cortical regions are related with the perturbation when multiple tasks are performed in parallel. As such, cortical sources corresponding to the onset of a whole-body perturbation are diverted in the presence of increased cognitive demand from mostly frontoparietal areas to include other parietal, as well as temporal and occipital sources.

Interestingly, during the single task involving postural recovery alone, cortical sources were predominantly estimated within the left hemisphere; whereas in the dual task it was found predominantly in the right hemisphere (Table 1). Serrien et al. (2006) discuss shifts in the dominance of the two hemispheres during different tasks, and note that the left hemisphere is considered to be dominant for motor behavior in right-handers. However, they also state that lateralization of motor function is indeed flexible and driven by several factors. The authors (Serrien et al., 2006) suggest the right hemisphere specializes in spatial functions, including spatial attention, and propose that the relative involvement of each hemisphere in a task depends on that task’s specific characteristics. In the current study, adding the secondary VWM task to the primary motor task (reactive postural control), could shift activity from the left to the right hemisphere, as the task shifted from primarily motor (left hemisphere) to one which also involved visual-spatial attentional processing (right hemisphere).

In summary, the present study estimated that the pre-, primary and supplementary motor areas—albeit to a lesser extent for the dual task—and somatosensory areas are primary cortical sources related to reactive postural control. For the single task, the cortical sources were mainly located within the frontal and parietal areas; whereas for the dual task, cortical sources included the frontal and parietal lobes and shifted to other locations including the temporal and occipital lobes. There was also a reduction in the mean absolute N1 ERP peak amplitude in the dual compared with single task. Although biased towards females, the current findings indicate that when attention is divided by performing a VWM task, resources within the brain are re-allocated in relation to the onset of a whole-body perturbation. Thus, we emphasize here that reactive postural control involves distinct electrocortical dynamics depending on the situation (i.e., single vs. dual task).

## Author Contributions

MDB contributed to the drafting, revising, analysis and interpretation of the data, and approval of the work and is accountable for all aspects of the work. PIB contributed to the analysis, interpretation of data, critical revision and approval of the work and is accountable for all aspects of the work. CEL contributed to the acquisition, interpretation of data, critical revision and approval of the work and is accountable for all aspects of the work. MHW and BHD contributed to the conception, design and interpretation of the data of the work. MHW and BHD revised, approved and is accountable for all aspects of the work.

## Funding

BHD was supported by a New Investigator Grant from the Medical Research Foundation of the Oregon and Health Sciences University Foundation.

## Conflict of Interest Statement

The authors declare that the research was conducted in the absence of any commercial or financial relationships that could be construed as a potential conflict of interest.

## Footnotes

^1^http://sccn.ucsd.edu/eeglab

^2^http://sccn.ucsd.edu/wiki/MPT
